# Born to Code: Does the Portrayal of Computer Scientists as Geniuses Undermine Adolescent Youths' Motivational Beliefs?

**DOI:** 10.3389/fpsyg.2021.709427

**Published:** 2021-08-06

**Authors:** Christine R. Starr

**Affiliations:** School of Education, University of California, Irvine, Irvine, CA, United States

**Keywords:** academic achievement, motivation, self-concept, stereotyped attitudes, intelligence

## Abstract

**Introduction:** Computer scientists are often stereotyped as geniuses who are naturally gifted in pSTEM (physical Sciences, Technology, Engineering, and Math). Prior correlational research found that this genius stereotype is negatively related to students' pSTEM motivation. However, the effect has not been explored experimentally to evaluate possible casual influences. Using situated expectancy-value theory as a framework, the present experiment tested whether media invoking the genius stereotype negatively impacts high school students' expectancy and value beliefs regarding pSTEM.

**Methods:** The sample comprised of 213 U.S. high school students (53% boys, 46% Asian). Participants read one of two versions of an article profiling a student majoring in computer science. The genius condition emphasized the student's natural talent and the importance of being gifted for success in computer science, whereas the control condition did not mention these attributes. Pre- and post-test measures of pSTEM expectancy and value beliefs were assessed.

**Results and Conclusions:** Students in the genius condition, but not the control condition, demonstrated a significant decline in pSTEM value beliefs. There was no effect on expectancy beliefs. Thus, popular stereotypes of persons in pSTEM as geniuses may contribute to students devaluing of pSTEM subjects. These stereotypes may be especially threatening to girls and students from minoritized backgrounds. Implications are discussed, including the need to address genius stereotypes in pSTEM classrooms.

This study experimentally tested the impact of a stereotypical portrayal of computer scientists as geniuses on high school students' motivational beliefs in physical sciences, technology, engineering, and mathematics (pSTEM). Researchers and policymakers have sought to increase students' interest in pSTEM fields given their importance in society [National Science Foundation (NSF), 2019]. The present study focuses on computer science primarily because computer science majors have some of the highest paid and most widely available job prospects among the pSTEM fields [National Science Foundation (NSF), 2019]. Yet, they continue to have low enrollment among women and other historically marginalized groups, even when compared to other pSTEM domains [National Science Foundation (NSF), 2019]. One reason for this may be the “coding genius” stereotypes that individuals often associate with computer science (e.g., Cheryan et al., 2013). Popular media representations of computer scientists (and other pSTEM professionals) as nerdy White men who are geniuses may steer some students away these fields (Cheryan et al., 2013). Moreover, these effects may be stronger among girls and women (Cheryan et al., 2013; Starr, 2018). Adolescence is an important period to consider the impact of stereotypes on pSTEM motivation because youth are exploring their identities and future selves (Lauermann et al., 2017; Wang et al., 2017).

Professionals in pSTEM fields are often stereotyped as geniuses who are brilliant or naturally gifted (Cheryan et al., 2013). In contrast to this fixed or essentializing stereotype, a growth mindset emphasizes that success in a discipline like pSTEM can be attained through effort (e.g., Dweck and Yeager, 2019). Prior work has found evidence for genius stereotypes regarding pSTEM among adolescents (Hannover and Kessels, 2004; Garriott et al., 2017; Starr and Leaper, 2019) and young adults (Storage et al., 2016; Ehrlinger et al., 2018; McPherson et al., 2018; Sáinz et al., 2019). For example, one study indicated that U.S. undergraduates were more likely to attribute success to brilliance in STEM fields than many other fields (Storage et al., 2016). This stereotype may influence youths' stereotypes about who belongs in pSTEM (Braden, 2020) and may negatively relate to motivational beliefs in pSTEM (Hannover and Kessels, 2004; Cheryan et al., 2013; Garriott et al., 2017; Starr, 2018). For example, one study among adolescents found that endorsement of the genius stereotype about pSTEM professionals was negatively related to pSTEM expectancy and value beliefs among those who did not see themselves as naturally gifted in pSTEM (Starr and Leaper, 2019). Mass media, including online news articles, may be one way of transmitting stereotypes, including the stereotypes about pSTEM professionals (Cheryan et al., 2013). However, to date no study has experimentally explored whether media invoking the genius stereotype negatively impacts pSTEM motivational beliefs.

Situated expectancy-value theory (Eccles and Wigfield, 2020) posits that motivational beliefs and the sociocultural context shape individuals' academic choices and achievement. Expectancy beliefs comprise individuals' ability self-concepts and expectations for future success in a subject, whereas value beliefs reflect their attainment value and perceived extrinsic utility of a subject. According to the theoretical model both expectancy and value beliefs are influenced by the cultural milieu (such as stereotypes present in the media), in addition to individual factors (such as gender) (Eccles and Wigfield, 2020). Extensive support for the theory has been established in many samples, including with U.S. adolescent samples (see Schoon and Eccles, 2014; Wigfield et al., 2015; Eccles and Wang, 2016). In the present study, exposure to materials emphasizing the genius stereotype about a computer science major was hypothesized to lead to a decrease in high school students' pSTEM expectancy and value beliefs. Students' gender was additionally tested as a potential individual moderator.

Genius stereotypes about pSTEM may have a greater negative effect on girls' pSTEM motivation for at least two reasons. First, prior studies suggest that people tend to view boys and men as more brilliant than girls and women (Bian et al., 2018). In addition, girls and women may be more likely to have self-concepts discrepant with stereotypes about persons in pSTEM fields (Ehrlinger et al., 2018; Starr and Leaper, 2019). Furthermore, the stereotype of people in pSTEM as geniuses frequently goes along with a related stereotype that they are nerdy (Starr, 2018; Starr and Leaper, 2019).

The present study sought to replicate and extend an earlier experiment that Cheryan et al. (2013) conducted with undergraduates. When participants read a newspaper article emphasizing the nerdy stereotype about computer scientists, undergraduate women expressed lower interest in computer science than those in a control condition (undergraduate men were not impacted). The current experiment extends this earlier work in three ways through its focus on high school students, its testing the impact of the genius stereotype, and its assessment of changes in both value and expectancy beliefs. In addition, high school students' gender was tested as a potential individual moderator. We chose to focus the experiment on a computer science major primarily because computer science majors have some of the highest paid and most widely available job prospects among the pSTEM fields [National Science Foundation (NSF), 2019]. Yet, these majors continue to have low enrollment among women and other historically marginalized groups, even when compared to other pSTEM domains [National Science Foundation (NSF), 2019]. One reason for this may be the “coding genius” stereotypes that individuals often associate with computer science (e.g., Cheryan et al., 2013).

## Method

### Participants

Participants were 213 students enrolled in physical science classrooms in four Northern California High Schools; 226 participants began the survey however 13 did not complete it. A power analysis indicated that 212 participants were needed to determine within-group differences. Participants were randomly assigned into the control condition (*n* = 110, 51.6%) and the experimental condition (*n* = 103, 48.4%). About half of participants self-identified as a girl (*n* = 100, 46.9%) and half as a boy (*n* = 113, 53.1%). Most of the students in the study were sophomores (*n* = 101, 47.4%) or juniors (*n* = 84, 39.4%). Additionally, there were 27 seniors (12.7%) and one first-year student. Students' self-identified ethnic-racial backgrounds included Asian (*n* = 100, 47%), White (*n* = 64, 30%), Latina/o/x (*n* = 25, 12%), multiethnic (*n* = 21, 10%), Black (*n* = 1), Native American (*n* = 1), or unreported (*n* = 1). Due to the small numbers of students from underrepresented groups, underrepresented status was not tested as a moderator.

### Procedure

After obtaining Institutional Review Board (IRB) approval, teachers were recruited via the school district science coordinator, and science teachers then recruited students in their classroom; all students were currently enrolled in at least one science and one math course. In a school district of seven high schools, four participated with about 4% of the 8,000 students completing the survey. Teachers gave the online survey to students in their classroom and were compensated for their time with a $100 Amazon gift card. After students assented, they were first asked demographic questions. Next, pSTEM (physical science, computer technology, engineering, math) was defined as such, with examples of pSTEM courses (e.g., chemistry, computer science, physics) (see Supplementary Materials for more detail). Students were then administered scales to assess expectancy-value beliefs in pSTEM. Next, students silently read one of the two randomly assigned short articles. Finally, students completed measures of their expectancy-value beliefs again. The expectancy-value scale items were presented in random order in the pre- and post-test. The experiment was the last portion of a larger study investigating nerd-genius stereotypes and self-concepts. Demographic variables were asked at the very beginning, followed by the 20-min survey (not included in the present study). Then, the experiment took place in the final 10 min. Participants answered the expectancy-value beliefs scale questionnaire, read the randomly assigned stimuli, then answered the post-experiment questions.

### Materials

**Figure 1:**
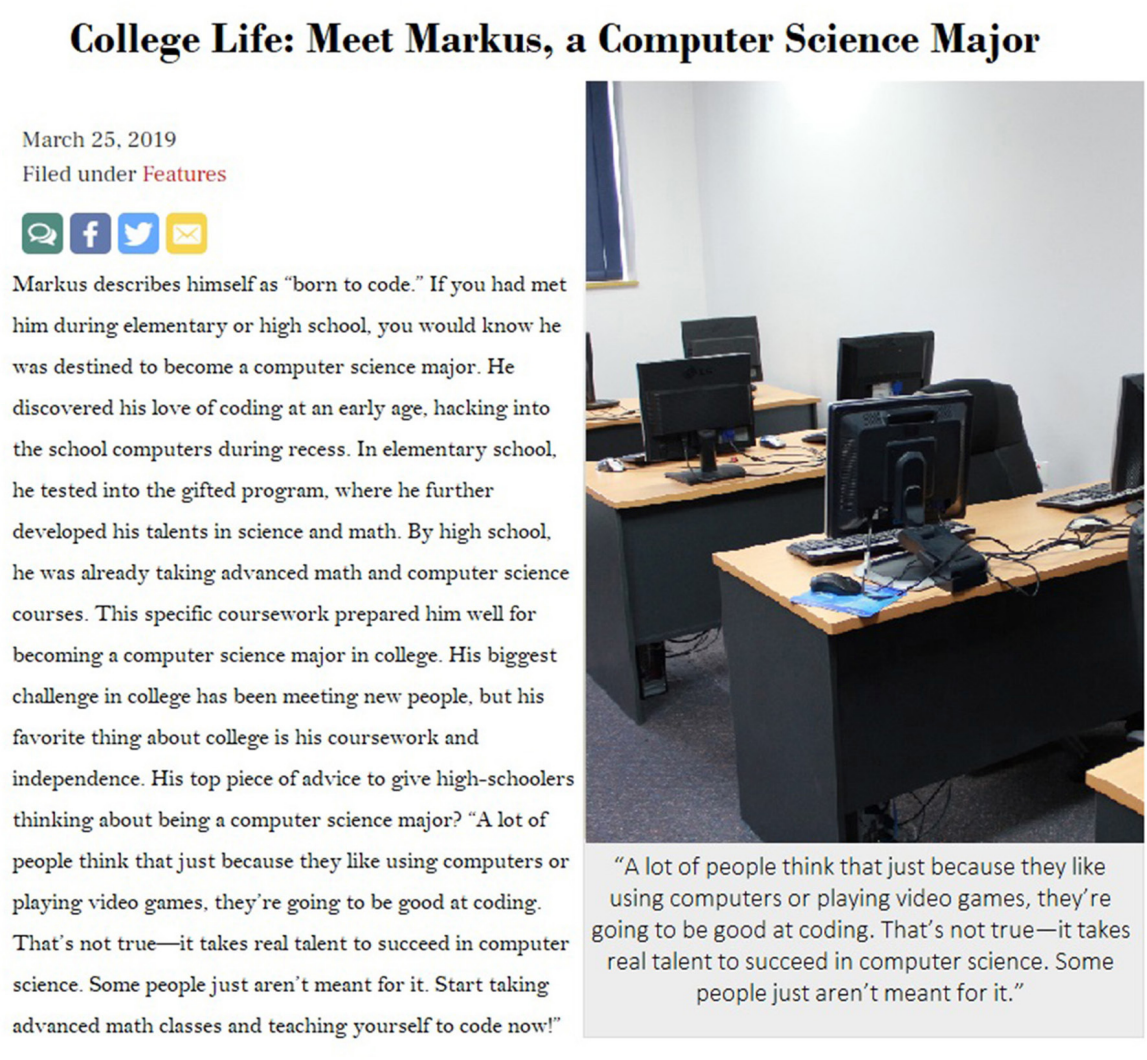
Genius experimental condition stimuli.

**Figure 2:**
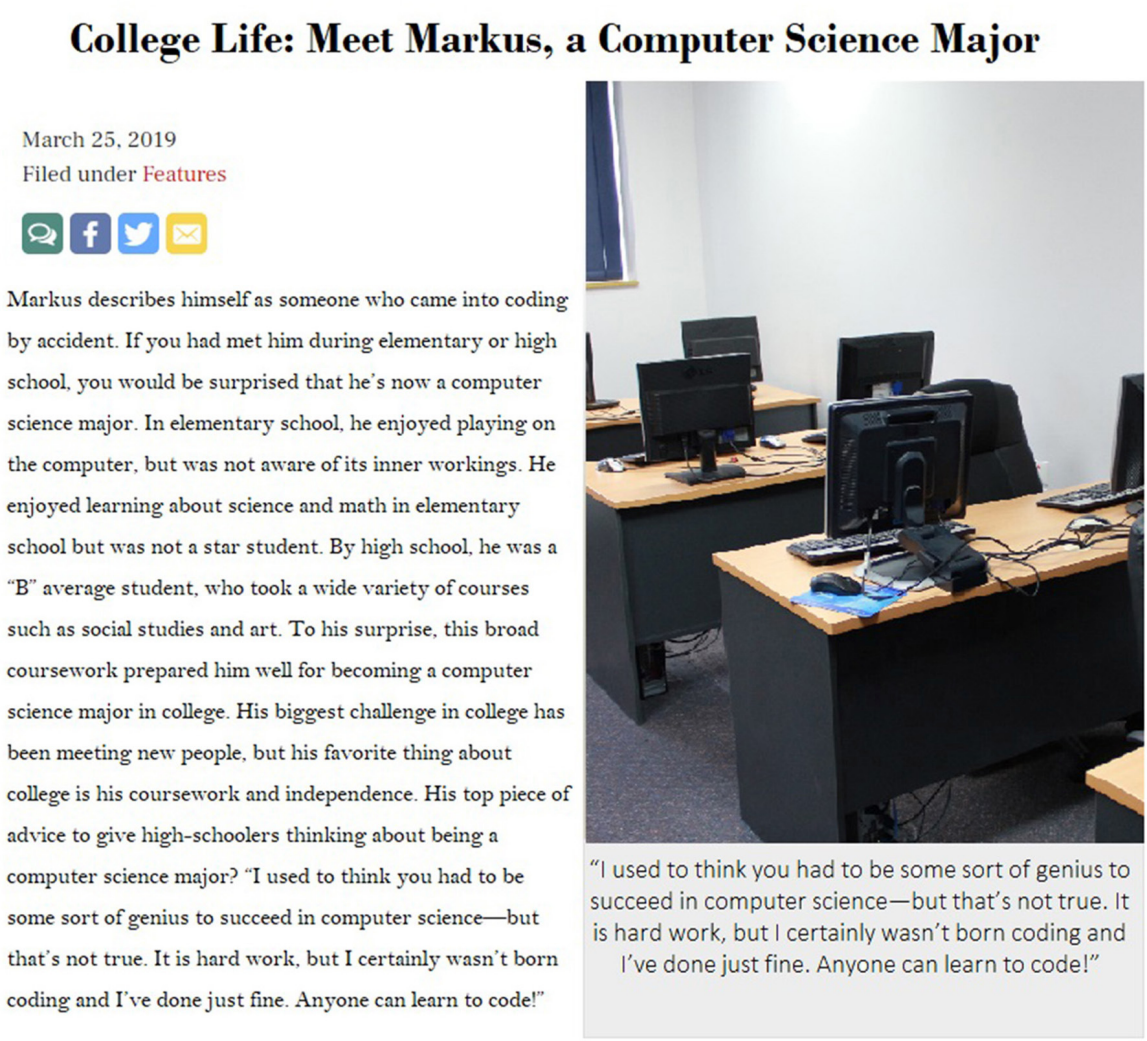
Control condition stimuli.

Participants were randomly assigned to read one of two fabricated high school newspaper articles created for this study. Both articles were entitled “College Life: Meet Markus, a Computer Science Major,” and discussed what college is like as a computer science major via a student named Markus. The length of the story was based on prior research (Cheryan et al., 2013) and the style of the mock online article (such as the font, social media logos, picture, and quotation style) mimicked the layout of the high school districts' online student paper. The genius condition article was written to invoke the genius stereotype about computer scientists (e.g., Cheryan et al., 2013). It portrayed Markus as someone who is naturally gifted in STEM subjects, began coding at an early age, and spent both his in-school and free time devoted to activities such as “hacking” and taking pSTEM courses. Additionally, Markus is quoted as describing coding as requiring “real talent” and stating that in order to succeed in college as a computer science major, high school students should solely focus on taking computer science courses and teaching themselves to code (see Figure 1 for full text). The control condition article portrayed Markus as a student who discovered coding later in life, took a variety of courses in high school (including art), and described coding as something “anyone can be good at” (see Figure 2 for full text). The control article directly challenged the genius stereotype with a quote from Markus (“I used to think you had to be some sort of genius to succeed in computer science, but that's not true”). Otherwise, the articles were similar and the same length.

### Measures

The measures used in the present study are described below (see Supplementary Materials for a full list of measures).

#### Background Variables

Students were asked to report their gender, ethnic/racial background, year in school, and math grade.

#### pSTEM Expectancy and Value Beliefs

Expectancy-value beliefs (Eccles and Wigfield, 1995) in pSTEM were measured before and after reading the article. Ten items assessed expectancy beliefs (pSTEM self-concepts and expectations for success; pretest: α = 0.92; posttest: α = 0.94). Example expectancy beliefs item: “How well do you expect to do in your pSTEM courses this year?” (1 = *not at all well*; 3 = *fairly well*; 5 = *exceptionally well*). Four items assessed value beliefs (attainment value and utility value; pretest: α = 0.86; posttest: α = 0.85). Example value beliefs item: “How important is it to you to do well in pSTEM courses?” (1 = *not at all important*; 3 = *somewhat important*; 5 = *most important*). All items were rated on a 5-point scale; this was chosen to give participants a neutral option (3) with two options on either side. Items were averaged in SPSS to create a scale. These scale items are frequently used in research implementing expectancy-value theory, and have been validated in prior studies, including with adolescent U.S. samples (Eccles and Wigfield, 1995; Lauermann et al., 2017). Measures met statistical assumptions for inferential testing (e.g., both skewness and kurtosis were between 1 and −1, Mauchly's Test of Sphericity was not significant).

## Results

Bivariate correlations were run across key variables (see Table 1). *T*-tests were conducted to assess potential experiment condition differences; conditions did not significantly differ in pretest pSTEM value or expectancy beliefs, math grade, gender, race/ethnicity, or year in high school (all *p'*s > 0.05).

**Table 1:** Bivariate correlations across major variables.

	**1**	**2**	**3**	**4**	**5**	**6**	**8**	**9**
1. Genius condition	–	0.03	0.03	−0.10	0.00	0.03	0.03	0.01
2. Value beliefs pre		–	0.49^**^	0.76^**^	0.54^**^	0.00	0.36^**^	0.13
3. Expectancy beliefs pre			–	0.50^**^	0.91^**^	−0.02	0.37^**^	0.12
4. Value beliefs post				–	0.56^**^	−0.01	0.37^**^	0.12
5. Expectancy beliefs post					–	−0.03	0.49^**^	−0.12
6. Girl						–	0.07	−0.07
8. Math grade							–	−0.08
9. Year in high school								–

** *p <0.01*.

To test the hypothesis that reading an article highlighting persons in pSTEM as geniuses would lower students' pSTEM expectancy and value beliefs, two mixed-design repeated-measures ANCOVAs were conducted. Condition (genius vs. control) and gender were between-group factors while math grade was controlled as a covariate. The repeated measures (Time) comprised pretest and posttest pSTEM value beliefs in one model and pretest and posttest expectancy beliefs in another model.

**Figure 3:**
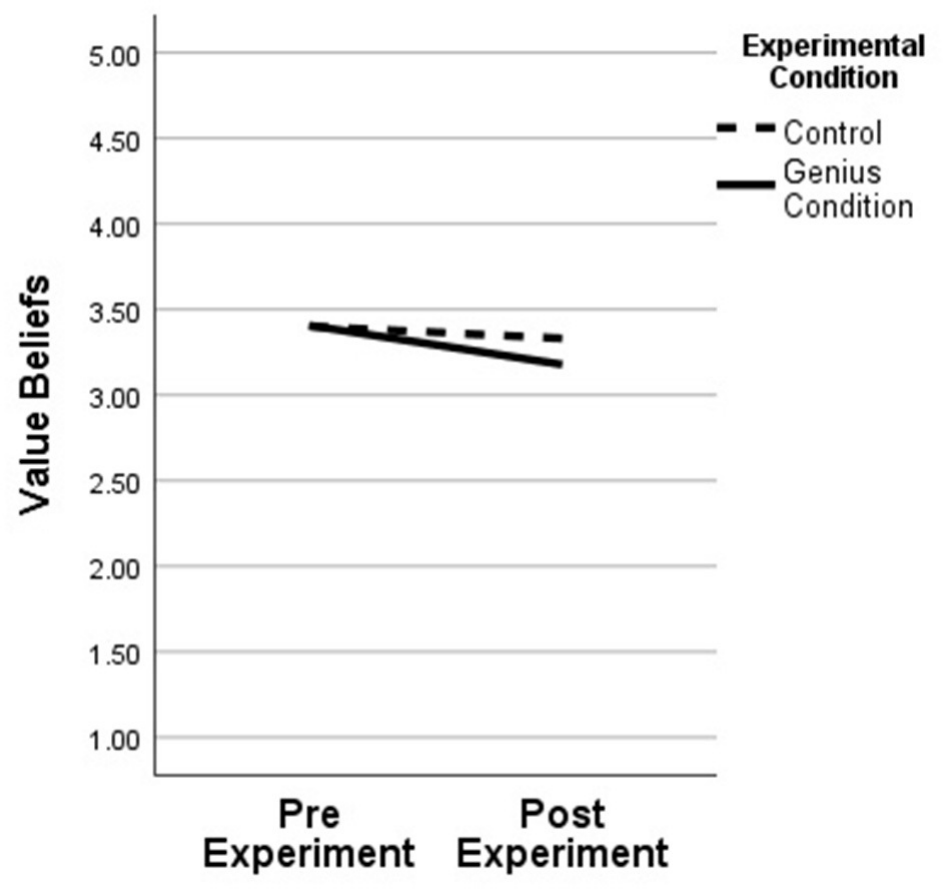
Value beliefs pre- and post- experiment, by condition. Controlling for student math grade.

As we hypothesized, there was a significant Condition x Time effect for pSTEM value beliefs (see Table 2). pSTEM value beliefs of students in the genius condition had lowered significantly following the experiment (pretest *M* = 3.41, *SD* = 0.81; posttest *M* = 3.19, *SD* = 0.83) when compared to those in the non-genius condition (pretest *M* = 3.40, *SD* = 0.78; posttest *M* = 3.33, *SD* = 0.68); [*F*_(1, 210)_ = 4.53, *p* = 0.03, η*p*^2^ = 0.021] (see Figure 3). However, there was not a significant Condition x Time effect with pSTEM expectancy beliefs; [*F*_(1, 210)_ = 1.01, *p* = ns, η*p*^2^ = 0.005]. Gender did not significantly moderate the interaction in either ANCOVA.

**Table 2:** Condition by time by gender ANCOVA.

	**Genius** ***M (SD)***			**Control** ***M (SD)***		
	**Girls**	**Boys**	**All**	**Girls**	**Boys**	**All**
Value Beliefs Pre	3.36 (0.79)	3.46 (0.83)	3.41 (0.81)	3.47 (0.85)	3.33 (0.72)	3.40 (0.78)
Value Beliefs Post	3.11 (0.77)	3.25 (0.88)	3.19 (0.83)	3.39 (0.77)	3.27 (0.60)	3.33 (0.68)
Change (Post-Pre)	−0.25	−0.21	−0.22	−0.08	−0.06	−0.07
**Source**	**SS**	**df**	**MS**	**F**	***p***	***ηp**^**2**^*
Condition x Time	0.651	1	0.651	4.532^*^	0.034	0.021
Condition x Time x Gender	0.006	1	0.006	0.041	0.840	0.000
Error	30.188	210	0.144			

*Controlling for math course grade*.

* *p <0.05*.

## Discussion

Using situated expectancy-value theory as a framework, this study investigated whether a short article portraying a computer science major as a genius (vs. hardworking) lowered high school students' expectancy and value beliefs. As hypothesized, students' pSTEM value beliefs significantly declined in the genius condition but not the control condition. This result complements a previous experiment with undergraduate women finding the nerd stereotype about computer scientists undermined their computer science interest (Cheryan et al., 2013). The findings support a tenet of situated expectancy-value theory that cultural stereotypes impact motivational beliefs. Moreover, the results are consistent with prior correlational research indicating negative relations between endorsing STEM-genius stereotypes and participants' STEM value beliefs (e.g., Hannover and Kessels, 2004; Garriott et al., 2017; Starr, 2018). The present study provides experimental evidence that genius-stereotyped media can reduce adolescents' value beliefs in the physical sciences, technology, engineering, and math.

In the present experiment, there was no effect on students' pSTEM expectancy. This finding is in contrast to prior correlational studies which found negative relations between genius stereotype endorsement and expectancy beliefs (e.g., Starr, 2018). Changes in expectancy beliefs may be more dependent on feedback and evaluation from others over time (Muenks et al., 2018). Prior research investigating the nerd stereotype about computer scientists did not investigate expectancy beliefs (Cheryan et al., 2013). Future work might examine both the nerd and genius stereotype's impact on pSTEM expectancy beliefs experimentally in different ways (e.g., repeated exposure, exposure combined with feedback).

Contrary to expectation, gender was not a significant moderator. In an earlier experiment upon which the present study was based, reading a stereotyped article about pSTEM professionals as nerdy indicated an effect on women but not men (Cheryan et al., 2013). However, research guided by balanced identity or prototype-matching approaches suggests the effect of the pSTEM-genius stereotype may partly depend on whether individuals view themselves as talented in the subject or whether they endorse the stereotype (e.g., Hannover and Kessels, 2004; McPherson et al., 2018; Starr and Leaper, 2019). In similar ways, these processes need to be explored with underrepresented minoritized students who may be less likely than other students to view themselves as geniuses. Future research might explore gender and race/ethnic as moderators among larger samples, using the balanced identity or prototype matching approach.

A few limitations of this study can be noted. First, the sample was not sufficiently large and ethnically diverse to analyze race/ethnicity as a potential moderator. Second, the experimental manipulation employed only a male character in each condition. Future studies should take into account the gender of the character. Furthermore, the manipulation was brief; future studies might longitudinally explore stereotyped media exposure influences motivational beliefs over time. Finally, the present study explored motivational beliefs in pSTEM, although prior research suggests that identity beliefs or belonging in pSTEM may also be affected by genius stereotypes. Future research might experimentally explore identity or belongingness outcomes and might include different types of media.

In conclusion, the present experiment highlights the importance of fostering an inclusive classroom climate that does not invoke or reinforce the genius stereotype (Cheryan et al., 2013). For example, classroom artifacts (e.g., posters of Einstein), popular media (such as *The Big Bang Theory*), and perhaps teachers' comments may highlight the genius stereotype about pSTEM. Furthermore, researchers can explore interventions to decrease the impact of this stereotype in conjunction with a growth mindset approach (e.g., Burnette et al., 2020).

## Data Availability Statement

The raw data supporting the conclusions of this article are available by the author at https://osf.io/uyfdr.

## Ethics Statement

The studies involving human participants were reviewed and approved by University of California, Santa Cruz Institutional Review Board. Written informed consent to participate in this study was provided by the participants' legal guardian/next of kin.

## Author Contributions

The author confirms being the sole contributor of this work and has approved it for publication.

## Conflict of Interest

The author declares that the research was conducted in the absence of any commercial or financial relationships that could be construed as a potential conflict of interest.

## Publisher's Note

All claims expressed in this article are solely those of the authors and do not necessarily represent those of their affiliated organizations, or those of the publisher, the editors and the reviewers. Any product that may be evaluated in this article, or claim that may be made by its manufacturer, is not guaranteed or endorsed by the publisher.

## References

[B1] Bian L. Leslie S. Murphy M. C. Cimpian A. (2018). Messages about brilliance undermine women's interest in educational and professional opportunities. J. Exp. Soc. Psychol. 76, 404–420. 10.1016/j.jesp.2017.11.006

[B2] Braden S. (2020). “Scientists can't really talk to people”: unpacking students' metacommentary on the racialized and gendered science nerd trope. Int. J. Multicult. Edu. 22:87. 10.18251/ijme.v22i2.2439

[B3] Burnette J. L. Hoyt C. L. Russell V. M. Lawson B. Dweck C. S. Finkel E. (2020). A growth mind-set intervention improves interest but not academic performance in the field of computer science. Soc. Psychol. Personal. Sci. 11, 107–116. 10.1177/1948550619841631

[B4] Cheryan S. Plaut V. C. Handron C. Hudson L. (2013). The stereotypical computer scientist: gendered media representations as a barrier to inclusion for women. Sex Roles 69, 58–71. 10.1007/s11199-013-0296-x

[B5] Dweck C. S. Yeager D. S. (2019). Mindsets: a view from two eras. Perspect. Psychol. Sci. 14, 481–496. 10.1177/1745691618804166 30707853 PMC6594552

[B6] Eccles J. S. Wang M. T. (2016). What motivates females and males to pursue careers in mathematics and science? Int. J. Behav. Dev. 40, 100–106. 10.1177/0165025415616201

[B7] Eccles J. S. Wigfield A. (1995). In the mind of the actor: the structure of adolescents' achievement task values and expectancy-related beliefs. Personal. Soc. Psychol. Bull. 21, 215–225. 10.1177/0146167295213003

[B8] Eccles J. S. Wigfield A. (2020). From expectancy-value theory to situated expectancy-value theory: a developmental, social cognitive, and sociocultural perspective on motivation. Contemp. Educ. Psychol. 61:101859. 10.1016/j.cedpsych.2020.101859

[B9] Ehrlinger J. Plant E. A. Hartwig M. K. Vossen J. J. Columb C. J. Brewer L. E. (2018). Do gender differences in perceived prototypical computer scientists and engineers contribute to gender gaps in computer science and engineering? Sex Roles 78, 40–51. 10.1007/s11199-017-0763-x 29367799 PMC5756563

[B10] Garriott P. O. Hultgren K. M. Frazier J. (2017). STEM stereotypes and high school students' math/science career goals. J. Career Assess. 25, 585–600. 10.1177/1069072716665825

[B11] Hannover B. Kessels U. (2004). Self-to-prototype matching as a strategy for making academic choices. why high school students do not like math and science. Learn. Instr. 14, 51–67. 10.1016/j.learninstruc.2003.10.002

[B12] Lauermann F. Tsai Y. Eccles J. S. (2017). Math-related career aspirations and choices within Eccles et al.'s expectancy–value theory of achievement-related behaviors. Dev. Psychol. 53, 1540–1559. 10.1037/dev0000367 28639806

[B13] McPherson E. Park B. Ito T. A. (2018). The role of prototype matching in science pursuits: perceptions of scientists that are inaccurate and diverge from self-perceptions predict reduced interest in science career. Personal. Soc. Psychol. Bull. 44, 881–898. 10.1177/0146167217754069 29405846

[B14] Muenks K. Wigfield A. Eccles J. S. (2018). I can do this! the development and calibration of children's expectations for success and competence beliefs. Dev. Rev. 48, 24–39. 10.1016/j.dr.2018.04.001

[B15] National Science Foundation (NSF) (2019). Women, Minorities, and Persons With Disabilities in Science and Engineering. Washington, DC: National Science Foundation. Available online at: https://ncses.nsf.gov/pubs/nsf19304/ (accessed March 08, 2019).

[B16] Sáinz M. Martínez-Cantos J. L. Rodó-de-Zárate M. Romano M. J. Arroyo L. Fàbregues S. (2019). Young Spanish people's gendered representations of people working in STEM. A qualitative study. Front. Psychol. 10:996. 10.3389/fpsyg.2019.00996 31133933 PMC6514192

[B17] Schoon I. Eccles J. S. (2014). Gender Differences in Aspirations and Attainment: A Life Course Perspective. Cambridge, UK: Cambridge University Press. 10.1017/CBO9781139128933

[B18] Starr C. R. (2018). “I'm not a science nerd!”: STEM stereotypes, identity, and motivation among undergraduate women. Psychol. Women Q. 42, 489–503. 10.1177/0361684318793848

[B19] Starr C. R. Leaper C. (2019). Do adolescents' self-concepts moderate the relationship between STEM stereotypes and motivation? Soc. Psychol. Edu. 22, 1109–1129. 10.1007/s11218-019-09515-4

[B20] Storage D. Horne Z. Cimpian A. Leslie S. (2016). The frequency of “brilliant” and “genius” in teaching evaluations predicts the representation of women and African Americans across fields. PLoS ONE 11:17. 10.1371/journal.pone.0150194 26938242 PMC4777431

[B21] Wang M. Ye F. Degol J. L. (2017). Who chooses STEM careers? using a relative cognitive strength and interest model to predict careers in science, technology, engineering, and mathematics. J. Youth Adolesc. 46, 1805–1820. 10.1007/s10964-016-0618-8 27975183

[B22] Wigfield A. Eccles J. S. Fredricks J. A. Simpkins S. Roeser R. W. Schiefele U. (2015). “Development of achievement motivation and engagement,” in Handbook of Child Psychology and Developmental Science: Socioemotional Processes, Vol. 3, 7th Edn, eds M. E. Lamb, and R. M. Lerner (Hoboken, NJ: John Wiley & Sons), 657–700. 10.1002/9781118963418.childpsy316

