# Supplementary material for: Born to Code: Does the Portrayal of Computer Scientists as Geniuses Undermine Adolescent Youths' Motivational Beliefs?
Source: Front Psychol. 2021 Aug 6;12:709427. doi: 10.3389/fpsyg.2021.709427 (PMC8377157; doi:10.3389/fpsyg.2021.709427)
Supplement: Supplementary file 1 [file Table_1.DOCX]

**Supplementary Materials**

**Supplementary 1. Instructions and Scale Items.**

**Instructions: Explanation of pSTEM.**

The following disciplines (courses/careers) are considered part of pSTEM:

Astronomy

Astrophysics

Biochemistry

Bioengineering

Chemistry

Computer Engineering

Computer science

Engineering

Geology

Physics

Math (e.g., Algebra, Calculus, Geometry)

Robotics

**Expectancy Beliefs** **(Pre and Post Experiment)**

**Instructions:** The following questions ask how well you see yourself doing in pSTEM (physical Sciences, Technology, Engineering, and Math) courses.

1. Some students are better in one subject than in another. Compared to your other school subjects, how good are you in pSTEM courses?
2. How well do you expect to do in your pSTEM courses this year?
3. How well do you think you will do in future pSTEM courses?
4. How good are you at learning something new in pSTEM?
5. In general, how confident are you in your ability to do well in pSTEM courses?
6. In general, how well can you learn the most challenging material in pSTEM courses?
7. In general, how difficult are pSTEM tasks for you?
8. Compared to most other school subjects, how hard are pSTEM courses for you?
9. How much effort will you need to do well in pSTEM courses in the future?
10. How hard do you have to study for pSTEM tests to get a good grade?

**Value Beliefs (Pre and Post Experiment)**

1. How important is it to you to do well in pSTEM courses?
2. Compared to most of your other activities, how important is it to you that you are good in pSTEM courses?
3. How useful is what you learn in pSTEM courses for your life after you finish high school?
4. Compared to most of your other activities, how useful is what you learn in pSTEM courses?
